# A promising approach to steady-state fusion: High-temperature superconducting strong-field stellarator with precise omnigenity

**DOI:** 10.1016/j.xinn.2023.100537

**Published:** 2023-11-14

**Authors:** Guosheng Xu, Zhiyuan Lu, Dehong Chen, Baonian Wan

**Affiliations:** 1 Institute of Plasma Physics, Chinese Academy of Sciences, Hefei 230031, China

## Abstract

The stellarator has inherent advantages over the tokamak in achieving steady-state operation, especially due to its absence of disruptions and lack of need for current drive and the associated recirculating power. In recent years, there have been remarkable advances in the field of stellarator optimization, where precisely quasi-symmetric and precisely quasi-isodynamic magnetic configurations have been achieved with coils, allowing the neoclassical transport and energetic particle losses of stellarators to be reduced to levels comparable to those of tokamaks. At the same time, the development of high-temperature superconducting magnet technology will potentially double the magnetic field strength of stellarators. While these strong fields are expected to introduce new challenges, and while turbulent transport remains a common challenge for both stellarators and tokamaks, the combination of these physical and technological advances results in the expectation that stellarators will become a competitive approach to tokamaks for realizing steady-state fusion.

## Introduction

Nuclear fusion, as powers the sun, potentially offers an environmentally friendly, intrinsically safe energy source with an abundant fuel supply. The stellarator is currently the second most funded magnetic confinement fusion approach in the world (after tokamak) in terms of research and development (R&D) investment. The fusion triple product $nT\tau_{E}$ (ion density, ion temperature, and energy confinement time), $6.7\times 10^{19}\text{keV}\;sm^{-3}$, obtained in 2018 on the world’s largest superconducting stellarator, W7-X (in Germany, costing ∼1 billion euros, commissioned in 2015),^1^ is still lower than the highest triple product obtained on the JET tokamak, $4.7\times 10^{20}\text{keV}\;sm^{-3}$ and is two orders of magnitude below that expected in ITER, $7.4\times 10^{21}\text{keV}\;sm^{-3}$, and DEMO, $∼1\times 10^{22}\text{keV}\;sm^{-3}$. Other magnetic confinement fusion approaches remain far from tokamak and stellarator in terms of the fusion triple product. Stellarator was invented earlier than tokamak, but its development has followed closely behind tokamak, and it has been considered to be the “spare tire” of tokamak. China’s magnetic confinement fusion research has been mainly focused on the tokamak approach, partially because of the difficulty of manufacturing three-dimensional (3D) coils of stellarators, among other reasons. The stellarator has inherent advantages over the tokamak in achieving steady-state operation and reducing recirculating power. In recent years, significant progress has been made in both stellarator experiments and stellarator design. In particular, recent advances in configuration optimization indicate that the neoclassical transport and energetic particle loss of advanced stellarators can potentially be reduced to levels comparable to those of tokamaks,^2^^,^^3^^,^^4^^,^^5^^,^^6^ while turbulent transport remains a common challenge for both stellarators and tokamaks.^7^

## Advantages and disadvantages of stellarator

Compared to the tokamak, the stellarator has many advantages. (1) The stellarator avoids MHD instabilities and major plasma discharge disruptions with large electromagnetic forces in the structure of the device and energetic beams of runaway electrons caused by the large plasma current of the tokamak. Stellarators do not suffer from error fields as much as in tokamaks due to weakly stable current-driven kinks and tearing modes in the case of a high plasma pressure ratio (β) or low density. Plasma maintenance and stability require singularly little active control, eliminating the need for stabilizing shells or coils. With sufficient passive stability, a stellarator is of great benefit to improve the availability factor of a power plant. (2) Stellarator is not subject to most of the limitations imposed by tokamak steady-state operation because it does not need to maintain a fully noninductive current. Enormous external power is needed in tokamaks to maintain not only the plasma current but also the profiles of pressure and current for achieving a high bootstrap current fraction and steady-state operation. In contrast, the stellarator magnetic field that confines plasma and the rotational transform of the field lines are mainly generated by external 3D coils, eliminating the need for large toroidal current flowing in the plasma and thus the need for external current drivers, which greatly lowers recirculating power and cost. (3) Stellarator plasma density is not limited by the Greenwald density of the tokamak, which is associated with its plasma current, but by the Sudo limit or radiation limit, which theoretically can be extended to a higher density through increasing heating power. (4) Stellarators allow for higher pellet fueling efficiency than tokamaks, making it easier to achieve a high burn fraction and tritium self-sufficiency. (5) Stellarators can adopt magnetic island divertors or nonresonant divertors. Full detachment was achieved in W7-X with island divertors. Benefiting from the long connection lengths of the field lines, the magnetic shear inside the islands is rather small, leading to a much smaller pitch angle of field lines than for a poloidal divertor in tokamaks, causing stronger perpendicular heat transport, a wider heat deposition area, and lower peak heat fluxes, which facilitates power exhaust. (6) Because the structure of the confining magnetic field is sufficiently dominated by external currents rather than by currents internal to the plasma, stellarators can be designed computationally with far better reliability and physical predictability than any other fusion concept. Tokamaks have advanced by extending from one generation of experiments to another. Compared to this extrapolation based on empirical scaling laws, computational design has significant advantages due to avoiding the construction of many intermediate test devices. With the improvement of high-performance computing capability and the application of artificial intelligence technology to the field of stellarator optimization, the advantages of computational design for accelerating the development of stellarators will become even more significant.

Stellarators also have some disadvantages.^8^ (1) The high precision requirements for 3D superconducting coils make them difficult to manufacture and install. (2) The more complex geometry makes for relatively poor accessibility to the plasma vessel and more challenging design of coil support structure, leading to challenges of blanket geometry designs and remote maintenance in stellarator reactors. (3) A strong temperature pedestal is not typical of H-modes in stellarators, leading to only mild improvements in confinement (∼20%) and to a more benign edge-localized mode activity than tokamaks. (4) The exhaust concepts in stellarators, including the magnetic island divertor, are far less mature than those of the poloidal divertor in tokamaks, e.g., an inherent weakness of the island divertor is the sensitivity of the strike-line position to plasma currents, which modifies the divertor neutral pressure, making density control difficult.

## Progress in stellarator research

Early stellarators, such as the large helical device (LHD) built in Japan in 1998 (a superconducting stellarator costing 50 billion yen, which has achieved more than 1 h continuous operation), have a much larger ripple in the magnetic field than tokamaks, resulting in significantly higher levels of plasma neoclassical transport and energetic particle loss than tokamaks. These lost energetic particles of future fusion reactors cause intolerable damage to the first wall and make it difficult to achieve self-sustaining burning, which is the main concern for achieving fusion using a stellarator. To address this issue, magnetic field configuration optimization and coil design have been the focus of research in the field of stellarators. In principle, the stellarator can be optimized to achieve an omnigenous property close to that of a tokamak, requiring that the contours of the magnetic field strength |B| on the flux surface can form continuous closed curves. The Lagrangian L for guiding-center drift motion, when expressed in Boozer coordinates, only depends on B, not the vector components of B. Consequently, an ignorable coordinate in B gives rise to a conservation law. The conserved quantity resembling canonical angular momentum ensures that each particle can only drift a distance on the order of a gyroradius away from a given flux surface, implying omnigenity. In the limit of perfect omnigenity, the bootstrap current vanishes, and neoclassical transport and energetic particle losses can be reduced to extremely low levels. There are two types of optimization approaches toward this goal: (1) quasi-isodynamic (QI) configuration,^5^^,^^6^ where the contours of the magnetic field strength |B| on the flux surface are poloidally closed, has the advantage of a low bootstrap current but usually has a higher aspect ratio (A). W7-X belongs to this category.^1^ (2) Quasi-symmetric (QS) configuration,^2^^,^^3^^,^^4^ according to the symmetry direction, is divided into quasi-axisymmetry (QA; e.g., NCSX device at Princeton Plasma Physics Laboratory, Princeton, NJ, USA, ceased construction), quasi-poloidal (QP) symmetry (e.g., QPS device at Oak Ridge, TN, USA, not built), and quasi-helical (QH) symmetry (e.g., HSX device at the University of Wisconsin, Madison, WI, USA). The QA configuration devices have been designed in Japan and France, but neither of them has been built. China’s Southwest Jiaotong University and Japan’s National Institute for Fusion Science (NIFS) collaborated in 2017 to design CFQS, the first copper-conductor QA stellarator in China, with construction expected to be completed by 2024.

W7-X demonstrated that an optimized advanced stellarator can reduce the neoclassical transport level below the gyro-Bohm turbulent transport level in the 1/ν low-collisionality regime,^1^ which is considered sufficient for a reactor, indicating that neoclassical transport is no longer a major weakness of stellarators. W7-X uses 50 3D coils and 20 planar coils. The high accuracy requirements and difficulty of manufacturing and mounting 3D coils with complex structures is a major challenge for the development of stellarators. As a result, only two countries have reported superconducting stellarators. However, after the manufacturing of W7-X, the worldwide technology of superconducting coil manufacturing has developed rapidly and has become more mature. The manufacturing of 3D superconducting coils is no longer a major limiting factor for the development of stellarators. After the technology matures, its cost will be comparable to that of manufacturing 2D coils.

## Recent advances in stellarator optimization

W7-X was designed in the 1980s. There remains opportunity for improved optimization beyond W7-X. In recent years, a series of advances have been made in stellarator optimization. First, in coil optimization, a direct optimization method for spatial 3D coils was developed.^9^ Furthermore, the optimization of the curvature, torsion, and cross-section of the coils was achieved, which makes the coil structure simpler and reduces the difficulty of manufacturing. The number of coils needed was also significantly reduced. A more important development is the recent finding that QA and QH configurations can be optimized to precise quasi-symmetry, i.e., with a tiny deviation from perfect symmetry, where neoclassical transport and energetic particle losses can be reduced to levels comparable to tokamaks for vacuum field^2^ and finite β.^3^ Immediately thereafter, it was demonstrated that these magnetic fields can be accurately produced using electromagnetic coils of only moderate engineering complexity.^4^ More recently, precise QI configurations of the W7-X type have also been shown to be achievable.^5^^,^^6^ These advances indicate that the previous major concerns about the stellarator approach, namely the neoclassical transport and the losses of energetic particles, can be addressed, representing a major breakthrough in the study of stellarator optimization.

However, we also note that these precise configurations are ideal designs. In reality, considering the error of coil manufacturing and installation and their vibration during stellarator operations, it is difficult to achieve such a high degree of precision, as well as the extremely low neoclassical transport and energetic particle losses. These recent advances are mainly in neoclassical optimization and do not address other issues, such as turbulent transport in stellarators. Even though the neoclassical transport can be reduced to acceptable levels through the optimization of the configuration, turbulent transport remains a common challenge for both stellarators and tokamaks, limiting the confinement performance, as demonstrated in W7-X, where relatively strong ITG-like turbulence appears in the case of ECRH electron-dominated heating, which leads to clamping of the central ion temperature to below 2 keV.^7^ W7-X has a relatively weak magnetic shear. Negative magnetic shear was observed to suppress turbulent transport on tokamaks and facilitate the stabilization of MHD instabilities. For the QA configuration, because the bootstrap current profile is off axis, a relatively strong negative magnetic shear forms spontaneously in the plasma core region, as shown in Figure 15 of Wechsung et al.^4^ A rotational transform (ι) profile with a relatively strong negative magnetic shear has been designed for the NCSX stellarator. Whether negative magnetic shear can also suppress turbulent transport on stellarators needs to be confirmed experimentally.

Among these optimized configurations, the precisely QI (PQI) configuration offers some advantages over the PQS class of configurations,^5^^,^^6^ i.e., the bootstrap current can be kept very small, and thus the configuration and the strike lines of magnetic island divertors do not change significantly with the plasma β, which is very important for achieving stable power exhaust and steady-state operations. This configuration and high-temperature superconducting (HTS) coils have been adopted by a stellarator fusion startup, “Proxima Fusion,” as the main technological route, which is the first spin out from the Max Planck Institute for Plasma Physics (IPP), founded on May 30, 2023, in Munich, Germany.

The PQA configuration is also a promising configuration that combines the advantages of a stellarator and advanced tokamak, enabling lower A values and making the device more compact.^2^^,^^3^^,^^4^ However, a more compact device requires larger neutron wall loads and divertor heat fluxes, which would increase the difficulty of implementing breeding blankets, applying neutron shielding to the coils, and managing divertor exhaust.^8^ A feature of the PQA configuration is that the neoclassical toroidal viscosity can be small and thus has the potential to generate a large toroidal rotation that can be utilized to suppress turbulent transport by rotational shear. A disadvantage of the PQA configuration is the lack of a validated divertor exhaust concept. The ι at the plasma boundary varies with the plasma current and β, and feedback control of the external current drive is needed to obtain a fixed boundary ι for stationary divertor exhaust.

**Figure 1:**
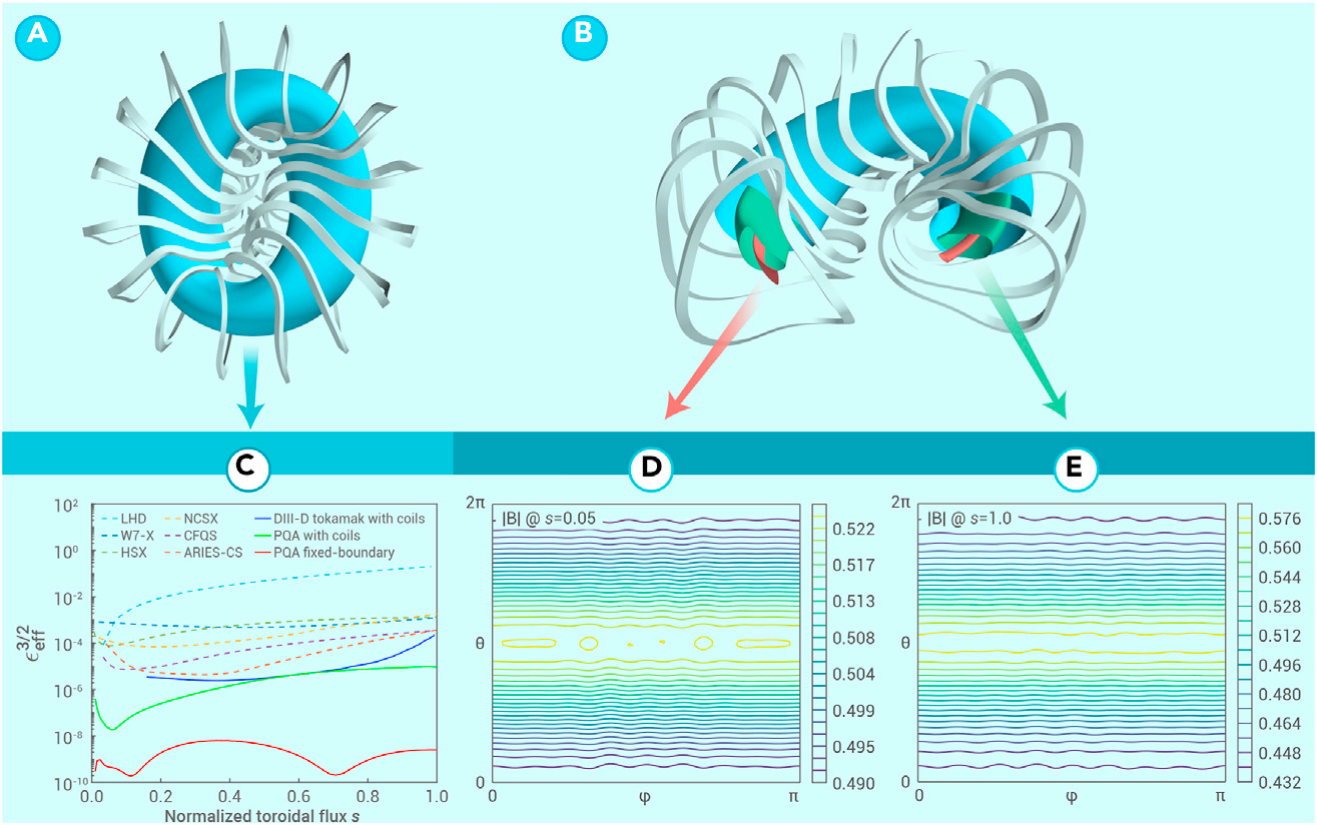
A stellarator design with PQA configuration (A) Top view. (B) Section view. (C) The neoclassical transport coefficients of the fixed-boundary equilibrium PQA and the free-boundary equilibrium PQA coils (produced by the coils) are much lower than those of conventional stellarators such as LHD, W7-X, HSX, NCSX, CFQS, and ARIES-CS and even lower than that of the DIII-D tokamak with ripples generated by coils. (D and E) Field strength |B| [T] produced by the PQA stellarator coils (D) on the *s* = 0.05 flux surface near the magnetic axis and (E) on the *s* = 1 flux surface at the plasma boundary.

For example, we provide a conceptual design of an HTS strong-field ($B_{0}=6T$) stellarator with a PQA configuration vacuum field produced by 16 module coils, with major radius *R*_0_ = 2*m*, toroidal periods $n_{fp}=2$, A=5, and mean ι=0.36, as shown in Figure 1. The highest magnetic field on the coil side reaches ∼17T, which is ∼70% of the highest magnetic field that can be achieved by an HTS coil. A configuration very close to PQA has been obtained, as demonstrated in Figures 1D and 1E by the field strength |B| contours produced by coils on the s=0.05 flux surface near the magnetic axis and on the s=1 flux surface at the plasma boundary, respectively. The neoclassical transport coefficients $\varepsilon_{eff}^{3/2}$ in the 1/ν regime computed by the *NEO* code for the PQA configuration are much lower than those of conventional stellarators and even lower than those of a tokamak with ripples (Figure 1C). If plasma density operates at the Sudo density limit $3.5\times 10^{20}m^{-3}$ with 11MW absorbed heating power, based on the ISS04 stellarator energy confinement scaling law,^8^ its fusion triple product $nT\tau_{E}$ can reach $∼2.8\times 10^{20}\text{keV}\;sm^{-3}$ with only a relatively small βof∼1.0% due to the strong field. Although this device is much smaller than W7-X, the fusion triple product is more than 4 times that of W7-X, mainly because of the strong magnetic field.

## Opportunities and challenges presented by HTS magnets

Technologically, magnets based on second-generation HTS tapes are becoming more mature, and the production capacity of these tapes is increasing rapidly. Moreover, the manufacturing technology of superconducting magnets in China has advanced greatly and is now ready to challenge the manufacturing of complex 3D HTS magnets, with which the magnetic field strength of stellarators is expected to double.^10^ Because $nT\tau_{E}\propto B^{4}R^{3}$,^8^ the volume size of a stellarator fusion reactor is expected to be reduced by nearly an order of magnitude. The HTS magnets are particularly suitable for the fully steady-state magnetic field of stellarators.^10^ Although the toroidal magnetic field (TF) of a tokamak is steady state, its TF magnets are still subject to a varying poloidal magnetic field, which limits the HTS magnet performance. In addition, the strong field enabled by HTS magnets lowers the plasma β and increases the tritium burnup fraction.^8^

While HTS strong fields may offer some attractive advantages, they also make engineering much more complicated and eventually limited by the forces produced by these fields, particularly the large stresses on the structural components.^8^ To date, it remains unclear whether the fields can be increased beyond the levels of previous power plant designs. In addition, modular coils, needed by optimized stellarators, certainly do not make this task easier. Moreover, a smaller wall surface leads to larger neutron wall loads and a faster degradation of in-vessel components, requiring more demanding breeding blanket technologies and maintenance schemes, which can severely limit the viability of small-size, high-field reactors.^8^ Furthermore, the unmitigated parallel heat fluxes toward the divertor are anticipated to grow with increasing magnetic field strength and decreasing device size.

## Summary

The tokamak remains the most globally invested and developed magnetic confinement approach, and it will likely be the first device to be built as a deuterium-tritium fusion reactor. However, tokamaks have inherent disadvantages compared to stellarators in achieving steady-state operation, especially under metal-wall conditions, mainly because of the high risk of plasma disruptions and the high cost of maintaining fully noninductive operating conditions. In recent years, with remarkable advances in the field of stellarator optimization and superconducting coil manufacturing technology, the two main concerns of early stellarators, namely large neoclassical transport and energetic particle losses, and the complex structure of 3D coils, which makes it difficult to manufacture and install, are expected to be significantly improved. In addition, HTS strong-field technology holds the promise of downsizing fusion reactors. The superposition of these physical and technological advances has the potential to make stellarators a very promising steady-state magnetic confinement approach for the development of fusion energy.
